# Antifungal effect of Algerian essential oil nanoemulsions to control *Penicillium digitatum* and *Penicillium expansum* in Thomson Navel oranges (*Citrus sinensis* L. Osbeck)

**DOI:** 10.3389/fpls.2024.1491491

**Published:** 2024-11-21

**Authors:** Merihane Gharzouli, Abdelhakim Aouf, Engy Mahmoud, Hatem Ali, Tawfiq Alsulami, Ahmed Noah Badr, Zhaojun Ban, Amr Farouk

**Affiliations:** ^1^ Laboratory of Applied Microbiology, Faculty of Natural and Life Sciences, University of Ferhat Abbas Setif1, Setif, Algeria; ^2^ Flavor and Aroma Chemistry Department, National Research Centre, Cairo, Egypt; ^3^ Food Technology Department, National Research Center, Cairo, Egypt; ^4^ Food Science and Nutrition Department, College of Food and Agricultural Sciences, King Saud University, Riyadh, Saudi Arabia; ^5^ Food Toxicology and Contaminants Department, National Research Centre, Cairo, Egypt; ^6^ Zhejiang Provincial Key Laboratory of Chemical and Biological Processing Technology of Farm Products, School of Biological and Chemical Engineering, Zhejiang University of Science and Technology, Hangzhou, China

**Keywords:** Thomson Navel oranges, antimicrobial, lemongrass oil, lemon oil, *Penicillium digitatum*, *Penicillium expansum*

## Abstract

Fungal infection is a potential issue in citrus fruits, while essential oils from *Cymbopogon citratus* and *Citrus limon* could be better alternatives to synthetic fungicides in orange preservation. The nanoparticles produced during ultrasonication exhibited a monomodal distribution of particle sizes with a mean zeta potential and a polydispersity index mean value of 74.12 nm, −38.4 mV, and 0.19 for *C. citratus* and 103 nm, −28.4 mV, and 0.22 for *C. limon*. The micrographs of the nanoemulsions exhibited spherical morphology with diverse nanometer-scale sizes. Nanoemulsification enhances the levels of neral and geranial in both oils while reducing the levels of limonene, γ-terpinene, and β-myrcene. The essential oils and their nanoemulsions exhibited good MIC values against Gram-positive and Gram-negative bacteria, ranging from 2% to 0.12%, while MBC was 4% to 0.25% (v/v) for both. The extended genetic investigation of the isolated fungal strains from Thomson Navel oranges through analysis of the ITS sequences and BLAST indicated 100% homology to those of *Penicillium digitatum* and *Penicillium expansum*. Both oils’ MIC and MFC values and nanoemulsions ranged from 0.12% to 0.06% and 2% to 0.03% against *P. expansum* and *P. digitatum*, respectively. Applying nanoemulsified *C. limon* and *C. citratus* as a coating on orange fruits significantly reduced the spread of *P. expansum* and *P. digitatum* fungi compared to the control. Coating with nanoemulsions reduced the negative changes in quality parameters during storage, such as weight loss, firmness, TSS, TA, pH, and ascorbic acid content. *Citrus limon* nanoemulsion did not alter the coated fruits’ sensory attributes compared to *C. citratus* nanoemulsion.

## 1 Introduction

Citrus fruits are one of the important crops economically for developing countries like Algeria and Egypt. In the 2023–2024 season, Egyptian orange production is expected to grow by 2.7% compared to the previous season, reaching 3.7 million tons, which accounts for 8% of the global production, according to the U.S. Department of Agriculture’s Foreign Agricultural Service (USDA, 2024). On the other hand, in 2021, the total production of citrus fruits in Algeria exceeded 1.5 million tons, consumed domestically and exported (FAO, 2021). Citrus fruits rot due to mechanical injury, tissue senescence, and microbial infection during harvest, storage, and transit, causing major economic losses. The most destructive citrus fruit diseases are *Penicillium digitatum* and *Penicillium expansum* fungi (Vilanova et al., 2012). Fungi are a significant pathogenic factor for fruit infection, as they can secrete mycotoxins, which threaten the liver, immunological, neurological, and fertility systems (Lima et al., 2021). A previous investigation reported the presence of toxigenic fungi infection, with a prevalence of *P. digitatum* (Rovetto et al., 2023). Furthermore, patulin and rubratoxin B mycotoxins were found in fruits after being infected by such fungi.

*Penicillium* was a significant post-harvest infection reported to cause global decay in citrus fruits. Most *Penicillium* investigations have recently focused on treatments against infection symptoms, fungicide resistance, and antagonist microorganisms (Costa et al., 2019). Chemical fungicides are used for control, but long-term usage causes resistance, food safety, and environmental damage (Wuryatmo et al., 2014). Safe and natural antistaling treatments are needed to reduce citrus diseases and preserve fruit quality. Antifungal resistance is a concern in agriculture as there have been reports of resistance to the main fungicide groups (Brauer et al., 2019). In this context, investigations are focused on finding natural and safe antifungal alternatives to synthetic chemicals. Recently, neoteric peptides were reported to have a significant ability to limit fungal development and reduce their genetic capacity to produce harmful metabolites (Lima et al., 2021). Furthermore, anti-mycotoxigenic and anti-mycotoxin production was reported to be positively affected by essential oils as natural antifungals.

Essential oils of aromatic plants contain bioactive compounds with antibacterial and antioxidant properties and reduced toxicity, recommended as possible synthetic fungicide replacements (Maghenzani et al., 2018). For instance, essential thyme, savory, and oregano oils suppress the toxigenic fungal species of *P. expansum*-infected orange and lime (Buonsenso et al., 2023). Again, cinnamon and carvone-rich oils were found to be active in reducing *P. expansum* contamination during orange storage and were reported to have fungicidal activity (Combrinck et al., 2011; Lai et al., 2021). Also, a significant inhibition by cinnamon, dill, *Origanum*, ylang-ylang, patchouli, vetiver, and chamomile oils was reported against the toxigenic fungi of orange (Zulu et al., 2023). Finally, essential oils of biobased black caraway and anise possessed effective inhibition against toxigenic fungi in blood orange fruit (Aminifard and Bayat, 2018).

Lemongrass and lemon essential oils are safe extracts with practical food applications that could be recommended as alternatives to synthetic antifungal chemicals. They contain antifungal volatiles, recommended for their potential post-harvest decay controls. Lemongrass oil exhibited antibacterial, antifungal, and anti-aflatoxigenic properties (Boudechicha et al., 2023). A significant antifungal activity against *P. digitatum* using ponkan mandarin citral was reported (Fan et al., 2014). Also, an *in-vitro* investigation showed that lemon and lemongrass oils positively affected *P. expansum* (Kgang et al., 2022). However, the essential oils’ short persistence, volatility, low solubility, and formulation difficulties restrict their use. Recent investigations are focused on their nanoemulsion applications to boost their utilization. Encapsulated essential oils in polymer matrixes inhibit *Aspergillus carbonarius*, increasing grape shelf life (Dhanasekaran et al., 2024). In the same line, *Citrus sinensis* L. oil nanoemulsion showed potential antifungal activity against *Aspergillus flavus*, *A. niger*, and *A. ochraceus* (Farouk et al., 2022), where its principal constituent, limonene, in collaboration with eugenol nanoemulsion, revealed the same on *Penicillium italicum* (Li et al., 2021).

Because of Egypt and Algeria’s economic dependence on citrus crops, this study examined the antibacterial and antifungal properties of lemongrass and lemon essential oils and their nanoemulsions against *P. digitatum* and *P. expansum* from decaying orange fruits. A low-energy preparation of coarse emulsion was followed by ultrasonication to create a nanoemulsion with Tween 80. We measured physicochemical factors for fruit quality. The pilot investigations on Thomson Navel oranges suggest using these nanoemulsions in edible coating films. In addition to the *in-vitro* nanoemulsion tests, the significant phytochemicals in both oils were docked with enzymes and target proteins involved in fungal metabolic processes to determine a possible mechanism of action for the antifungal effect.

## 2 Materials and methods

### 2.1 Chemicals and plants

All chemicals applied in this investigation were of HPLC grade and purchased from Sigma-Aldrich, Saint Louis, MO, USA. The lemon fruits, scientifically called *Citrus limon*, were supplied by a local market in Setif, Algeria. *Cymbopogon citratus* were collected from Bou Saada, 245 km south of Algiers, Algeria. Following identifications by a botanist at the Department of Biology and Plant Ecology, Setif-1 University, Algeria, a voucher specimen validating the plants’ identity was placed in our laboratory herbarium, and the Algerian numbers CAS09/01/23 (for *C. limon*) and CAS28/06/21 (for *C. citratus*) were assigned. Then, the collected plant material was air-dried at ambient room temperature in shaded conditions. Thomson Sweet oranges were purchased from a local Setif, Algeria market in December 2023.

### 2.2 Essential oil extraction

Essential oils were extracted using hydrodistillation with a Clevenger apparatus. Fresh peels from *C. limon* and leaves of *C. citratus* (100 g each) were boiled separately with distilled water for 3 h. The oil phase was condensed, and the essential oils were extracted, dried with anhydrous sodium sulfate, and stored in airtight glass vials sealed with aluminum foil at −20°C until analysis. This process was repeated three times (Boudechicha et al., 2023).

### 2.3 Nanoemulsion formulation

The initial step involved preparing a coarse emulsion by mixing the organic phase of the essential oil and a surfactant (Tween 80) (4:1; v/v) using a stirrer (37°C/2 h). Next, the organic phase was added drop by drop to double-distilled water while continuously stirring it using a magnetic stirrer. Then, the formed emulsion was subjected to ultrasonic emulsification using a probe sonicator (Ultrasonic Microprocessor VCX 500, Fisher Scientific, Loughborough, UK) with a power of 250 W. Before the ultrasonic treatment, the samples were chilled to 4°C and kept on ice throughout the sonication process (Patel and Ghosh, 2020).

### 2.4 Nanoparticle characterization

The stability of the nanoemulsions was checked by centrifugation (3,500 rpm/30 min) to observe the separation phase (Sigma 3-18KS, Osterode am Harz, Germany). The separation percentage was calculated as:


% Separation=(SAPW/TEW)×100

Where,

SAPW = separated aqueous phase weight and

TEW = total nanoemulsion weight.

The particle size distribution, polydispersity index (PDI), and ξ-potential of the nanoemulsion samples were measured using a Zetasizer Nano ZS (Nano-S90, Malvern Panalytical Ltd., UK) at a temperature of 25°C ± 0.1°C. Deionized water was used for dilution to prevent multiple scattering effects. The morphology of nanoemulsions was analyzed (Boudechicha et al., 2023) using transmission electron microscopy (TEM) at 160 kV operation (JEOL Ltd., Tokyo, Japan).

### 2.5 Gas chromatography-mass spectrometry

Gas chromatography-mass spectrometry (GC-MS) analysis reveals the effect of homogenization on the profile and constituents of essential oils. After mixing with diethyl ether, vortexing, and drying over anhydrous sodium sulfate, the nanoemulsion was transferred to a screw-cap vial for analysis. The extraction process was repeated thrice. Separation of the oils and nanoemulsion volatiles was carried out by using GC (Agilent 8890 System) coupled with an MS (Agilent 5977B GC/MSD) equipped with an HP-5MS capillary column (30 m, 0.25 mm i.d., 0.25 mm film thickness). The sample size injected was 1 µL at 230°C in a split mode (1:50). The oven was initially set at 50°C, raised at a rate of 5°C/min to 200°C and then from 200°C to 280°C at a rate of 10°C/min, and kept isothermal for 7 min. Mass spectra in electron EI mode were obtained at 70 eV, and the *m*/*z* ranged from 39 to 500 amu. Peaks were identified by comparing them to NIST, standards, and published data. Percentages of detected compounds were calculated using GC peak areas. Kovats index of each compound was determined using retention times of C_6_–C_26_ n-alkanes and compared to literature values (Himed et al., 2016; Boudechicha et al., 2023).

### 2.6 *In vitro* antimicrobial activity of essential oils and their nanoemulsions

#### 2.6.1 Microorganisms

The antibacterial activity was carried out on Gram-positive and Gram-negative bacterial strains procured from the Laboratory of Applied Microbiology at Ferhat Abbas University in Setif, Algeria. Gram-positive strains consisted of *Staphylococcus aureus* (ATCC 25923) and *Bacillus subtilis* (ATCC 6633). Gram-negative strains were *Escherichia coli* (ATCC 25922), *Salmonella typhimurium* (ATCC 14028), *Klebsiella pneumoniae* (ATCC 13883), and *Pseudomonas aeruginosa* (ATCC 27853). The evaluation of antifungal activity was performed on *A. niger* ATCC 16888, *A. flavus* ATIM 698, *Fusarium culmorum* KF91, and *Candida albicans* ATCC 10231 obtained from the National Research Centre, Egypt, in addition to isolated strains of *P. expansum* and *P. digitatum* retrieved from decaying orange fruits. The *Penicillium* strains were cultured on potato dextrose agar (PDA) medium at a temperature of 25°C/5 days; after purification and macroscopic and microscopic identification at the Laboratory of Applied Microbiology, Algeria, the fungi were identified through molecular means by Gene Life Science in Algeria.

#### 2.6.2 Molecular characterization

Genomic DNA was extracted from fungal mycelium using the NucleoSpin Plant II kit (Macherey-Nagel, Germany). PCR amplification targeted the ITS, Ef1, BT, and LSU rDNA regions. For ITS, primers ITS1 and ITS4 were used with an annealing temperature of 55°C to get fragments at the expected size of 600 bp (Gardes and Bruns, 1993). For Ef1, primers EF-728F and EF-2 were employed at a temperature of 52°C to amplify fragments at the predicted size of 450 bp (Carbone and Kohn, 1999). The PCR mixture included ultrapure water, Taq buffer (Promega, Madison, Wisconsin, USA), MgCl_2_ (25 mM), dNTPs (25 mM), forward and reverse primers (10 μM each), and Taq polymerase (Promega), and genomic DNA was added. Thermal cycling comprised an initial denaturation at 95°C for 5 min, followed by 35 cycles of denaturation at 95°C for 30 s, annealing at 55°C–52°C for 30 s, and extension at 72°C for 45 s, with a final extension at 72°C for 7 min. PCR products were visualized on a 1.5% agarose gel stained with GelRed (Biotium, USA) and imaged under UV light using a Bio-Rad Gel Doc system (USA). Purification of the PCR products was done using the NucleoSpin Gel and PCR Clean-up kit (Macherey-Nagel, Germany) and sequenced via the Sanger method (Sanger et al., 1977) using the BigDye v3.1 kit, with sequences analyzed using the ChromasPro software and compared to the GenBank database using the NCBI BLAST program for identification.

#### 2.6.3 Disk diffusion assay

The antimicrobial activity and nanoemulsions of the essential oils were evaluated using a slightly modified agar disk diffusion method (Micić et al., 2021). Bacterial and fungal suspensions were spread on agar plates. Paper disks soaked with essential oils and nanoemulsions were placed on the plates alongside positive controls (ceftriaxone and amphotericin B). After incubation, inhibition zones were measured. Each experiment was conducted in triplicate.

#### 2.6.4 Determination of minimum inhibitory concentration

The minimum inhibitory concentration (MIC) was determined using the microdilution method on a 96-well microplate following the CLSI, C. S. M (2018). Essential oils and nanoemulsions were diluted to achieve 4% to 0.01% v/v concentrations. The MIC for bacteria and fungi was evaluated using serial dilutions in a 96-well microtiter plate. The microplates were then incubated, and MICs were determined using tetrazolium chloride as a viability indicator for bacteria.

#### 2.6.5 Determination of minimum bactericidal and fungicidal concentrations

To determine minimum bactericidal (MBC) and minimum fungicidal (MFC) concentrations, an aseptic aliquot was collected from the cultures in wells with no visible turbidity, transferred to agar media, and incubated for 24 h at 37°C for bacteria and 5 days for fungi. All the tests were performed in triplicates (Boudechicha et al., 2023).

### 2.7 *In vivo* antifungal assay

With minor modifications, the technique of OuYang et al. (2020) was applied to disinfect fruits and artificially infect them with *P. digitatum* and *P. expansum* before the treatment with nanoemulsions. Orange fruits were selected based on size, color uniformity, and minimal surface injuries. These selected oranges were then subjected to disinfection using sodium hypochlorite solution (2%) for 2 min, followed by two washes with distilled water; the oranges were then left to air dry overnight. Subsequently, the oranges were divided into two treatment groups: T1 with *C. citratus* nanoemulsion and T2 with *C. limon* nanoemulsion, and a control group was treated with distilled water. Each treatment group was divided into four subgroups. In the first subgroup, oranges were treated with the respective nanoemulsions using the dipping method for 2 min.

In contrast, the second and the third subgroups were artificially infected with 15 μL of 10^5^ (spores/mL) sporal suspension of *P. digitatm* and *P. expansum*, respectively. After inoculation, the second and third subgroups were left to air dry for 24 h before being immersed in nanoemulsions. The fourth subgroup served as the control and was also wounded with a similar spore suspension of *P. digitatum* and *P. expansum*, along with treatment with distilled water. All treated orange fruits were stored in clear plastic boxes at room temperature for 21 days.

### 2.8 Evaluation of fruit quality

Five fruits were weighed at the beginning of storage and labeled as W1 for each treatment. These same fruits were reweighed after 21 days and marked as W2. The weight loss as a percentage was calculated using the following formula (Motamedi et al., 2018):


$$\mathrm{weight}\;\mathrm{loss}\;\left(\%\right)\;:\frac{\sum_{i=1}^{n}\frac{w1-w2}{w1}}{n}\;\times 100$$

The fruit samples were collected from each replicate and then juiced using a hand-press juicer. The total soluble solids (TSS, %), total acidity (TA, %), and pH were measured on both the first day (day 1) and the last day of 21 days of storage (Ncama et al., 2017). The TSS content of the juice was determined using a handheld manual refractometer, measuring in degrees Brix, and pH was measured using a pH meter. Simultaneously, TA was expressed as a percentage (%) of citric acid, measured via titration with 0.1 N of sodium hydroxide to reach pH 8.2, following the given formula:


$$\mathrm{TA}\left(\%\right)=\frac{\left(0.0064\right)\left(\mathrm{volume}\;\mathrm{of}\;\mathrm{NaOH}\;in\;\mathrm{ml}\right)}{10\mathrm{ml}\;\left(\mathrm{juice}\right)}\times \;100$$

Fruit firmness was determined for three fruits of each replication using a Brookfield CT3 Texture Analyzer (AMETEK Brookfield, Middleboro, MA, USA) fitted with a 3R probe. The iodometric titration method was used to determine the ascorbic acid content of fruit juice, which was expressed as the amount of ascorbic acid (mg) per 100 mL of juice (Motamedi et al., 2018).

### 2.9 Sensory analysis

On both day 1 and day 21, four coated oranges from each treatment and oranges from the control group that showed no fungal growth were chosen and sliced into several pieces for sensory evaluation. A group of 12 panelists from the Faculty of Natural and Life Sciences, University of Ferhat Abbas Setif1 in Setif, Algeria (10 men and 10 women), were selected to evaluate the quality attributes of the fruits, including color, aroma, flavor, and overall acceptance. This assessment was conducted using a scale with endpoints at 0 and 5. For the color, the range was from pale yellow (0) to dark orange (5), while aroma and flavor were rated on a scale ranging from weak (0) to vigorous (5). Ultimately, a hedonic scale was implemented to evaluate overall preference, ranging from dislike extremely (0) to like extremely (5), as previously referenced (Radi et al., 2018).

### 2.10 Statistical analysis

GraphPad Prism (GraphPad Software Inc., San Diego, CA, USA) and Microsoft Excel were used for statistical analysis and graphing. Results are shown as means ± standard deviations (SD) from three replicates. ANOVA and Tukey’s multiple range tests assessed differences between mean values, with *P* ≤0.05 indicating significance.

## 3 Results

### 3.1 Effect of nanoformulations on the compositions of essential oils

**Figure 1:**
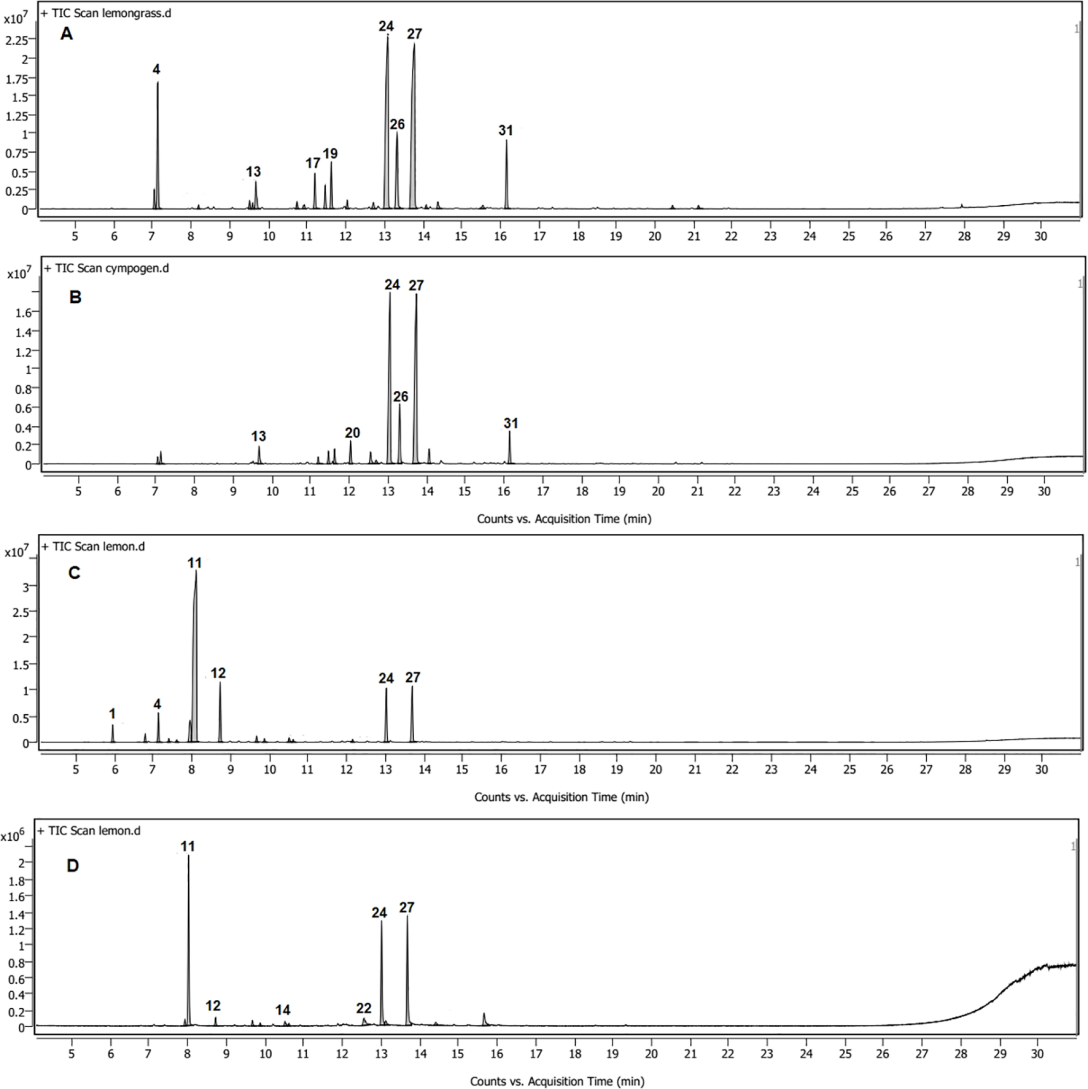
GC-MS chromatograms of *Cymbopogon citratus* oil **(A)** and its nanoemulsion **(B)** and *Citrus limon* oil **(C)** and its nanoemulsion **(D)**.

The yields of the hydrodistilled essential oils were 1.45% ± 0.04% and 1.25% ± 0.03% for *C. citratus* and *C. limon*. The identification of chemical composition using GC-MS revealed the presence of 21 constituents in *C. citratus* oil, representing 99.56% of the total volatile content (**Table 1**; **Figure 1A**). Neral and geranial were the predominant compounds, with 27.97% and 31.7%, respectively, followed by geraniol (8.49%), geranyl acetate (5.25%), and β-myrcene (9.64%). The nanoemulsion volatile pattern of the *C. citratus* oil showed the same trend with quantitative differences compared to the content of the raw oil (**Table 1**; **Figure 1B**). The same major components of the oil were identified as predominates in the nanoemulsion extract, except for β-myrcene, which dropped dramatically to 1.29%. The drop in the concentrations of many monoterpenes of the nanoemulsion like β-myrcene, linalool, isoneral, and isogeranial and the absence of others such as ocimene and rose furan were accompanied by an increase in neral (33.58%), geranial (38.47%), and estragole (2.92%), compared to the raw oil. The identified components in the nanoemulsion were represented in 100% of the total extract, with only 14 constituents.

**Table 1:** Volatile constituents’ identification of *Cymbopogon citratus* and *Citrus limon* oils and their nanoemulsions using GC-MS.

S/N	Compound	RI^a^	LRI^b^	Area %				Identification^c^
				*C. citratus* oil	*C. citratus* nanoemulsion	*C. limon* oil	*C. limon* nanoemulsion	
1	α-Pinene	942	939	–	–	1.88	–	RI, MS, STD
2	Sabinene	978	975	–	–	0.92	–	RI, MS
3	6-Methyl-5-heptene-2-one	983	985	1.27	0.77	–	–	RI, MS
4	*β*-Myrcene	992	991	9.64	1.29	3.46	–	RI, MS, STD
5	Octanal	1,001	998	–	–	0.42	–	RI, MS
6	3-Carene	1,013	1,011	–	–	0.29	–	RI, MS
7	*Z-β-*Ocimene	1,040	1,037	0.25	–	–	–	RI, MS
8	*E-β-*Ocimene	1,051	1,050	0.57	–	–	–	RI, MS
9	Rose furan	1,063	1,065	0.42	–	–	–	RI, MS
10	*p*-Cymene	1,025	1,024	–	–	4.27	1.29	RI, MS, STD
11	D-Limonene	1,031	1,029	–	–	61.8	35.59	RI, MS, STD
12	γ-Terpinene	1,058	1,059	–	–	7.77	1.74	RI, MS, STD
13	Linalool	1,100	1,096	2.46	2.31	0.75	1.42	RI, MS, STD
14	*cis*-Limonene oxide	1,136	1,134	–	–	0.66	1.45	RI, MS
15	*trans*-Limonene oxide	1,143	1,142	–	–	0.33	0.63	RI, MS
16	Isocitral (exo-)	1,147	1,144	0.31	–	–	–	RI, MS
17	Isoneral	1,171	1,170	2.63	0.85	–	–	RI, MS
18	Rose furan oxide	1,180	1,177	1.64	1.48	–	–	RI, MS
19	Isogeranial	1,189	1,185	3.45	1.79	–	–	RI, MS, STD
20	Estragole	1,199	1,196	0.63	2.92	–	–	RI, MS, STD
21	Decanal	1,203	1,201	–	–	0.38	–	RI, MS
22	Rose ether	1,221	1,223	–	1.79	–	3.81	RI, MS
23	Citronellol	1,228	1,225	0.64	0.63	–	–	RI, MS
24	Neral	1,240	1,238	27.97	33.58	7.89	24.77	RI, MS, STD
25	Carvone	1,245	1,243	–	–	–	1.43	RI, MS, STD
26	Geraniol	1,258	1,255	8.49	8.36	–		RI, MS, STD
27	Geranial	1,270	1,267	31.7	38.47	8.75	27.15	RI, MS, STD
28	Anethole	1,279	1,283	0.26	1.73	–	–	RI, MS, STD
29	Neryl formate	1,284	1,284	0.68	–	–	–	RI, MS
30	Nerolic acid	1,337	1,340	0.47	–	–	–	RI, MS
31	Geranyl acetate	1,384	1,383	5.25	4.03	–	–	RI, MS
32	Caryophyllene oxide	1,582	1,583	0.3	–	–	–	RI, MS
33	Selin-6-en-4α-ol	1,633	1,636	0.31	–	–	–	RI, MS
	Total	–	–	99.56	100	99.57	99.28	–

a RI, retention indices were calculated using the DB-5 column using alkane standards.

b LRI, retention indices according to the literature.

c Confirmed by comparison with the retention indices, the mass spectrum of the authentic compounds, and the NIST mass spectra library data.

On the other hand, only 14 compounds were identified in the *C. limon* oil, accounting for 99.57% of the total volatiles extracted. Limonene (61.8%), geranial (8.75%), neral (7.89%), and γ-terpinene (7.77%) were the main constituents of the oil (**Table 1**; **Figure 1C**). Increases were observed in the neral and geranial of the nanoemulsion (24.77% and 27.15%) at the expense of limonene and γ-terpinene, which showed a significant decline to 35.59% and 1.74%, with the absence of many monoterpenes identified in the oil like α-pinene, sabinene, β-myrcene, and 3-carene (**Table 1**; **Figure 1D**).

### 3.2 Nanoparticle characterizations

**Table 2** presents the physical characteristics of the nanoemulsions formulated. The samples exhibited an average height ratio of 100%, indicating excellent stability with no apparent phase separation, creaming, or sedimentation. The nanoemulsions of *C. citratus* and *C. limon* had average particle sizes of 74.12 ± 2.33 nm and 103 ± 3.72 nm, respectively. They both exhibited a monomodal size distribution pattern, indicating their ultrafine sizes of less than 200 nm. The mean zeta potentials were −38.4 ± 1.45 mV and −28.4 ± 2.33 mV, as shown in **Table 2**. Additionally, the PDI demonstrated uniformity in dispersion, with mean values of 0.19 ± 0.04 and 0.22 ± 0.07 for both *C. citratus* and *C. limon* nanoemulsions (**Supplementary Figure S1**).

**Table 2:** Physical characterizations of the nanoemulsions.

Property	Value	
	*C. citratus* nanoemulsion	*C. limon* nanoemulsion
Refractive index	1.33 ± 0.004	1.472 ± 0.005
Density (g/mL)	0.96 ± 0.002	1.136 ± 0.005
pH	5.01 ± 0.008	5.47 ± 0.009
Stability (separation in mm)	ND	ND
Particle size (nm)	74.12 ± 2.33	103 ± 3.72
PDI	0.19 ± 0.04	0.22 ± 0.07
Zeta potential (mV)	−38.4 ± 1.45	−28.4 ± 2.33

ND, not detected.

**Figure 2:**
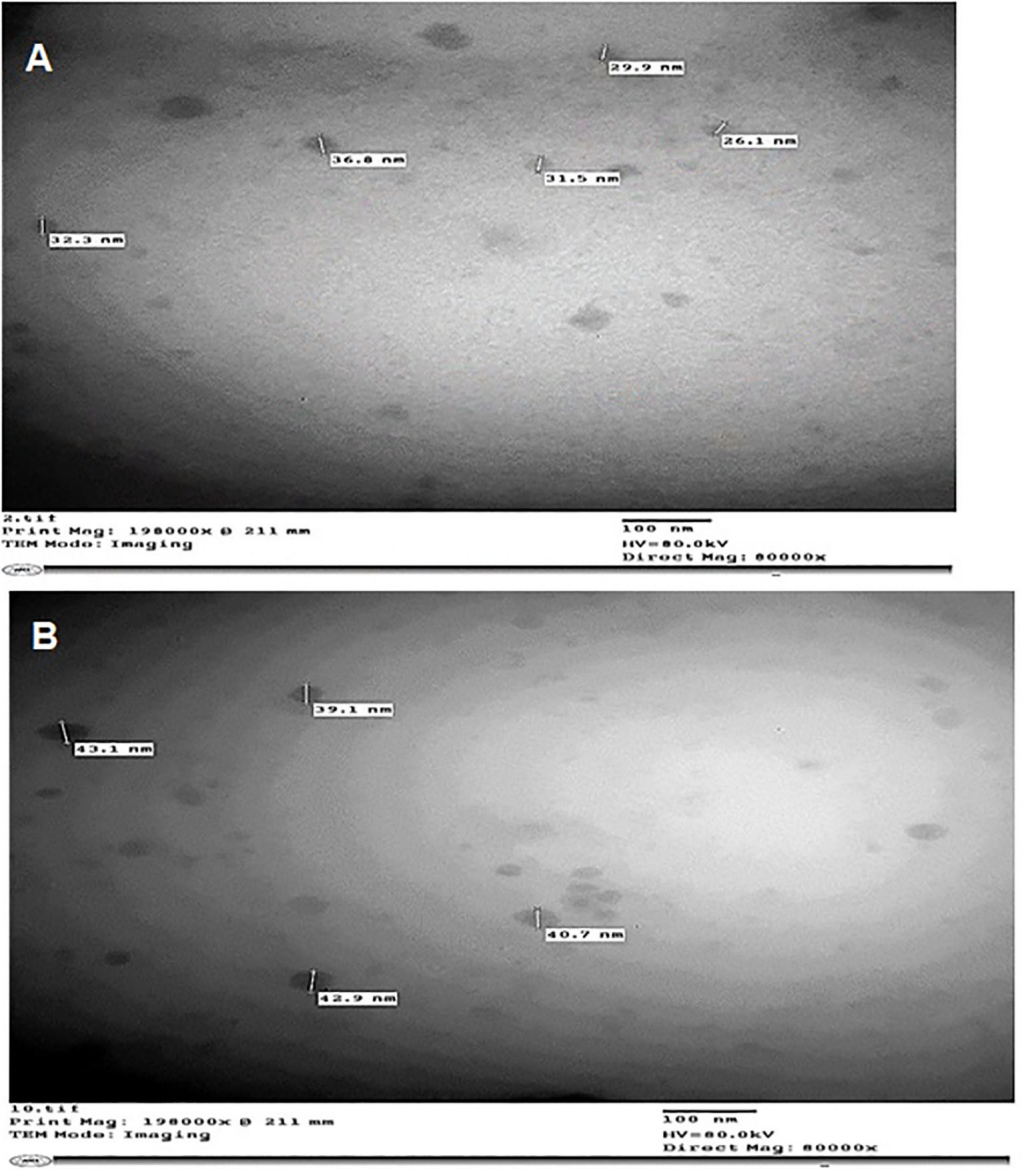
TEM micrograph of *C*. *citratus* **(A)** and *C*. *limon* **(B)** nanoemulsions.

TEM was used to characterize the nanoemulsions of *C. citratus* and *C. limon*. The droplets were evenly distributed and appeared to darken, indicating a successful preparation. TEM micrographs revealed spherical nanoparticles with varying diameters in nanometers (**Figures 2A, B**).

### 3.3 Morphological and molecular identification of fungal isolates

**Figure 3:**
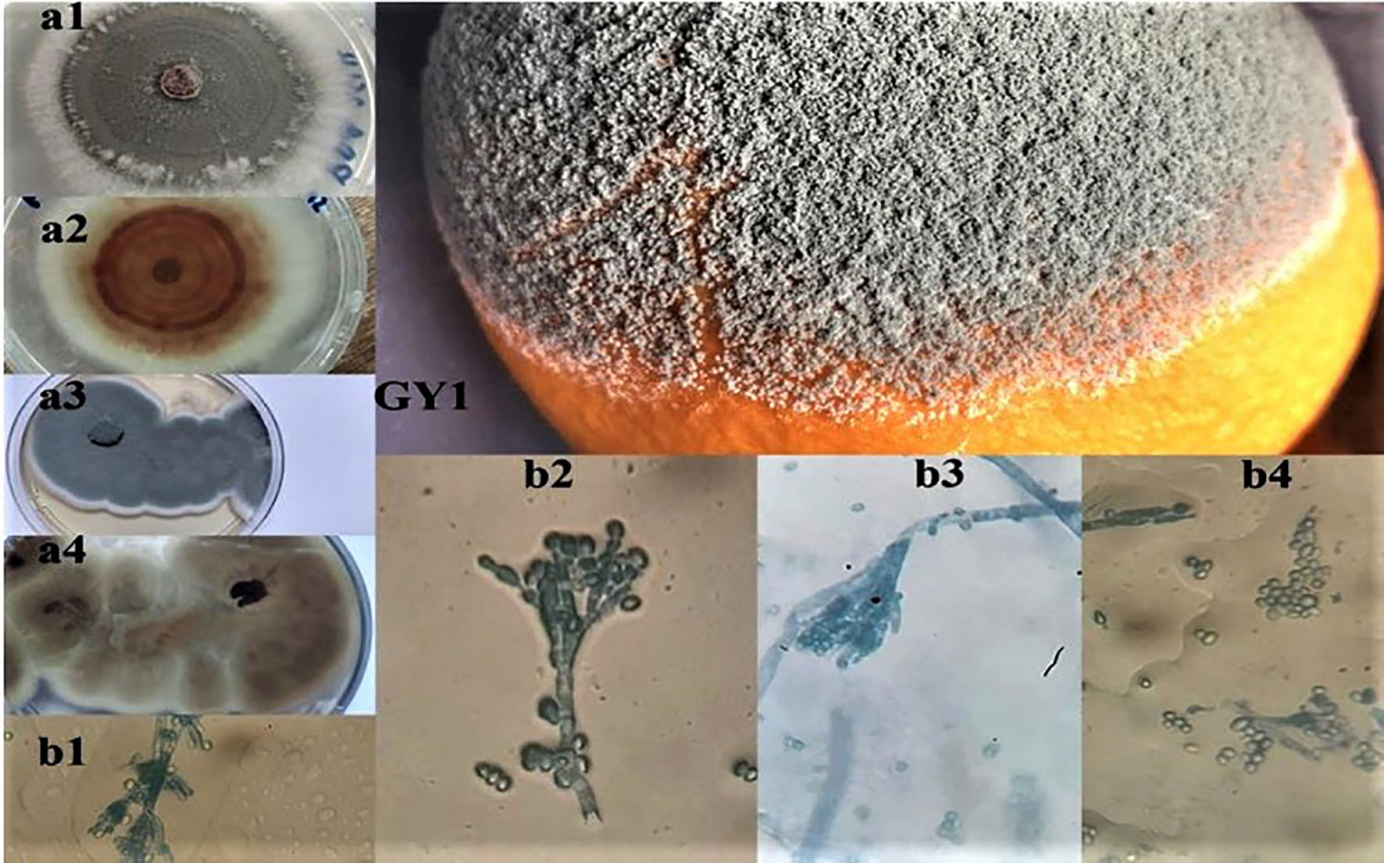
Macroscopic (a1, a2, a3, a4) and microscopic observations (b1, b2, b3, b4) of the isolated fungi (GY1) from decaying orange fruits, where (a1, a2) and (a3, a4) represent the aspect on PDA and Sabouraud media.

**Figure 4:**
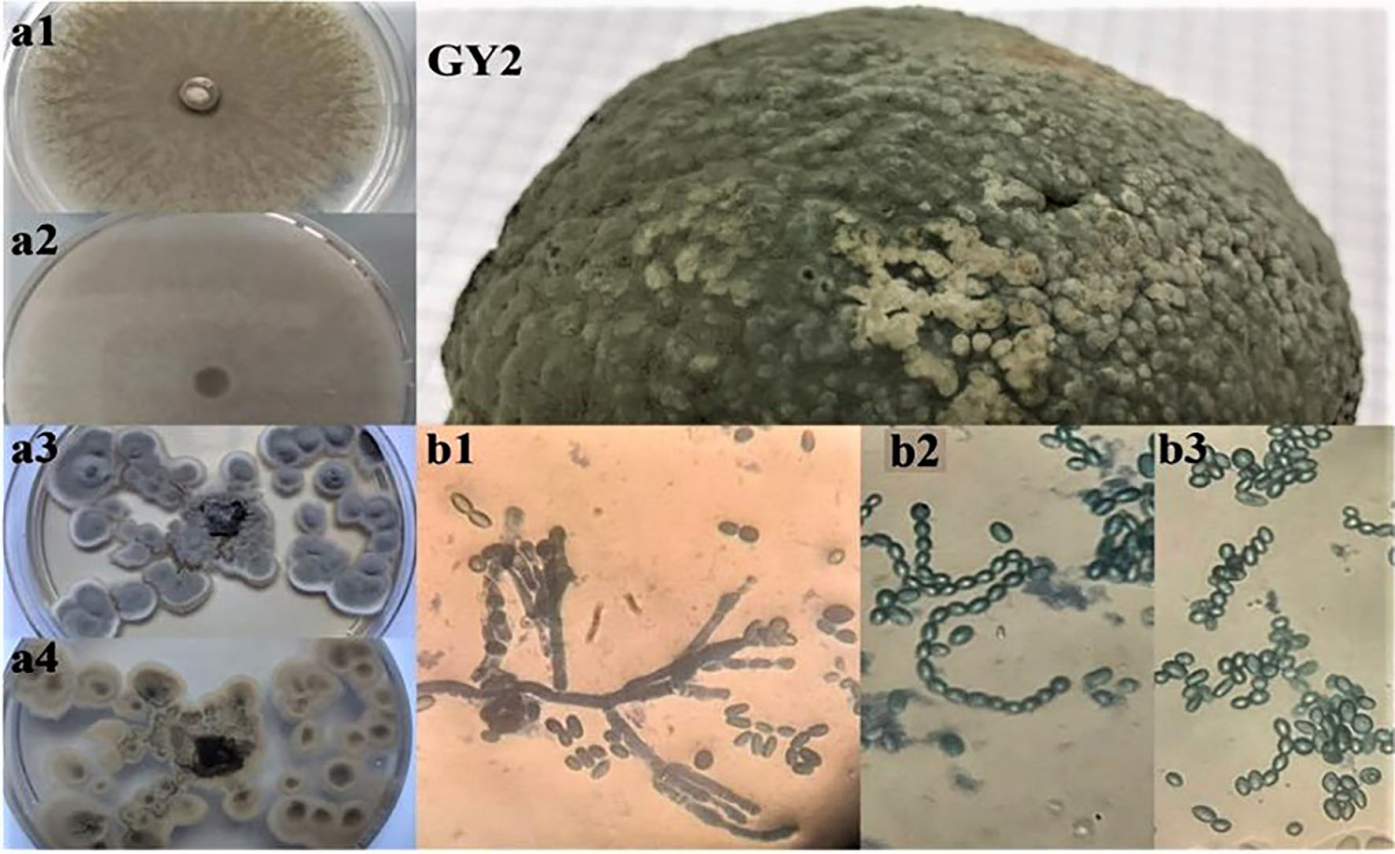
Macroscopic (a1, a2, a3, a4) and microscopic observations (b1, b2, b3) of the isolated fungi (GY2) from decaying orange fruits, where (a1, a2) and (a3, a4) represent the aspect on PDA and Sabouraud media.

A rigorous identification was undertaken upon macroscopic and microscopic examination of both fungal isolates obtained from orange fruits. It was found that both isolates belonged to the *Penicillium* genus based on their macroscopic features, such as color and consistency, along with microscopic observations of conidiophores and spores (**Figures 3**, **4**). This process ensured the accuracy of our findings. The genetic investigation of the isolated fungal strains, designated as GY1 and GY2, involved examining the ITS and TEF1 sections, which had lengths of 660 bp and 450 bp, respectively. The identification process involved doing a BLAST search against the GenBank database. The analysis of the ITS sequences indicated that strain GY1 displayed a 100% homology to both *P. italicum* and *P. expansum*, whereas strain GY2 showed a 99% similarity to *P. digitatum*. Further analysis using BLAST confirmed that GY1 corresponded to *P. expansum* (100% match), while GY2 was identified as *P. digitatum* (100% match).

### 3.4 *In vitro* antimicrobial activity of the oils and their nanoemulsions

#### 3.4.1 Antibacterial effect

**Figure 5:**
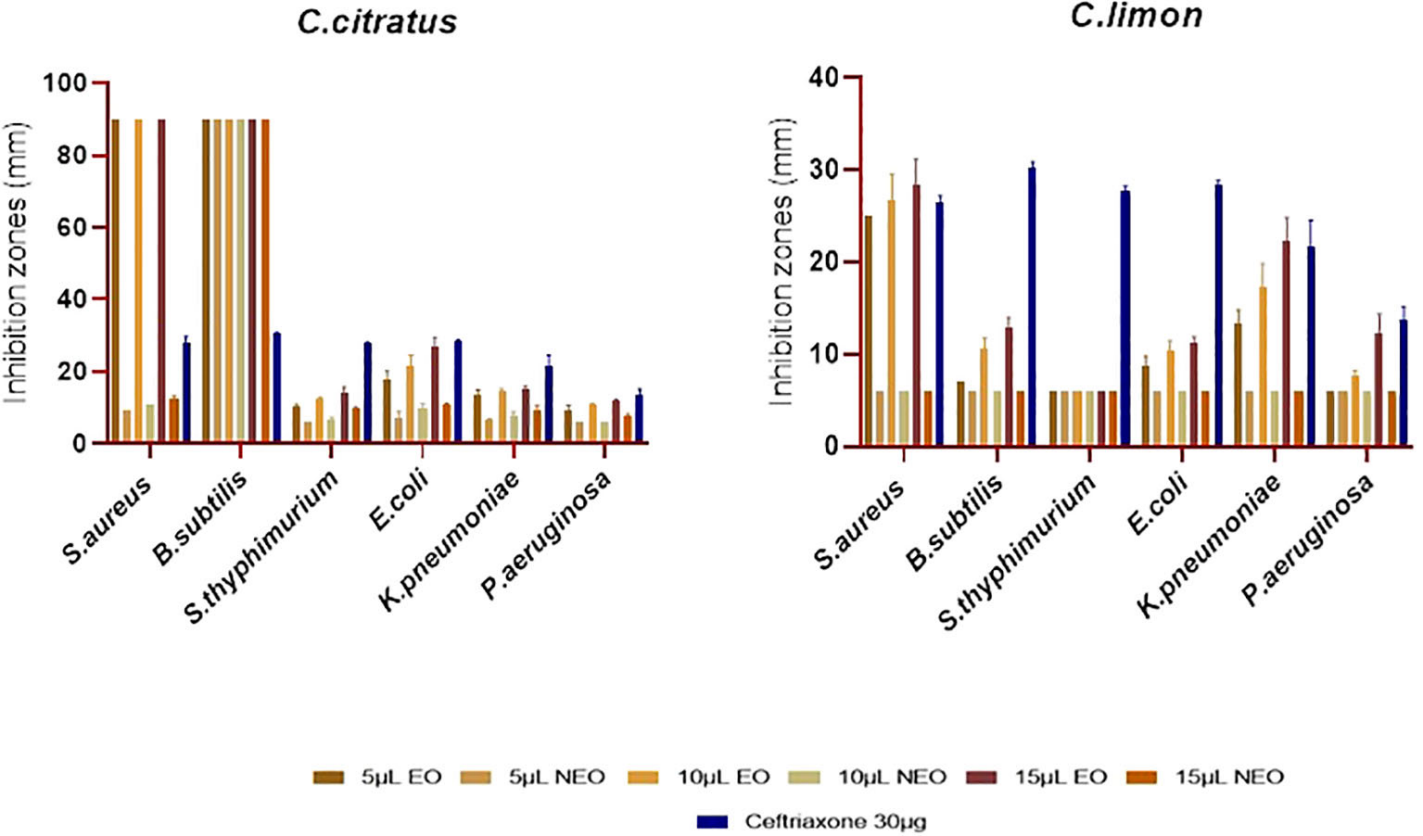
Antibacterial activity of *C. citratus* and *C. limon* essential oils and their nanoemulsions using disk diffusion assay, with ceftriaxone as the standard inhibitor, represented with error bars indicating ± standard deviation (*n* = 3).

The antimicrobial activity of the essential oils and their nanoemulsions was tested using both disk diffusion and microdilution methods. The aforementioned disk diffusion method showed that the tested essential oils and their nanoemulsions showed significant differences in antimicrobial activity against Gram-positive and Gram-negative bacteria (**Figure 5**).

The *C. limon* essential oil did not show activity against *P. aeruginosa* at 5 µL and 10 µL but exhibited moderate activity at 15 µL (*P* ≤ 0.05), resulting in a 12.33 ± 2.08 mm inhibition zone. Its activity against *E. coli* was weak, with inhibition zones ranging from 8.67 ± 1.15 mm to 11.33 ± 0.57 mm. *Citrus limon* oil was not active against *S. typhimurium* or *B. subtilis* at 5 µL. However, it showed weak to moderate activity at 10 µL and 15 µL (*P* ≤ 0.05) against *B. subtilis*, with inhibition zones of 10.66 ± 1.15 mm and 13 ± 1 mm. However, *C. limon* oil strongly affected *S. aureus*, with inhibition zones ranging from 25 ± 0 mm to 28.33 ± 2.88 mm (**Figure 5**). *Cymbopogon citratus* essential oil was the most active against all the tested strains in comparison with *C. limon*, especially against Gram-positive bacteria (*P* ≤ 0.05), where it completely inhibits the growth of *S. aureus* and *B. subtilis.* In contrast, *C. citratus* oil showed weak to moderate activity against *P. aeruginosa*, with inhibition zone diameters ranging from 9 ± 1.73 mm to 11.67 ± 0.57 mm. For *K. pneumoniae* and *S. typhimurium*, the activity of *C. citratus* oil was moderate, with diameters of the inhibition zone spanning from 13.33 ± 1.53 mm to 15.33 ± 0.58 mm and 10.33 ± 0.57 mm to 14 ± 1.73 mm, respectively. Regarding *E. coli*, the activity was strong, with inhibition zone diameters varying from 17.67 ± 2.51 mm to 26.67 ± 2.89 mm (**Figure 5**).

The current study used the microdilution technique to determine the MIC and MBC of the oils and their nanoemulsions. The results are presented in **Table 3**. The essential oils exhibited good MIC values against both Gram-positive and Gram-negative bacteria, with MIC values ranging from 2% to 0.12%. The microdilution method showed better results than the disk diffusion method. Consistent with the previous results from the disk diffusion method, *C. citratus* oil displayed the best MIC values of 0.12% against *E. coli* and *K. pneumoniae* and 0.25% against *S. aureus*, *B. subtilis*, and *P. aeruginosa.* The MIC values of *C. limon* oil were 0.5% against *S. aureus* and *P. aeruginosa*, 1% against *E. coli and K. pneumoniae*, and 2% against *B. subtilis* and *S. typhimurium* (**Table 3**).

**Table 3:** MIC and MBC determinations of *Cymbopogon citratus* and *Citrus limon* essential oils and their nanoemulsions against bacteria.

Strain	*C. citratus* oil		*C. limon* oil		*C. citratus* nanoemulsion		*C. limon* nanoemulsion	
	MIC (%)	MBC (%)	MIC (%)	MBC (%)	MIC (%)	MBC (%)	MIC (%)	MBC (%)
*S. aureus* ATCC 25923	0.25	0.25	0.5	0.5	0.25	0.5	–*	–
*B. subtilis* ATCC 6633	0.25	1	2	2	0.25	2	–	–
*S. typhimurium* ATCC 14028	0.5	2	2	2	2	4	–	–
*E. coli* ATCC 25922	0.12	0.25	1	1	0.25	2	–	–
*K. pneumoniae* ATCC 13883	0.12	0.25	1	1	0.25	2	–	–
*P. aeruginosa* ATCC 27853	0.25	2	0.5	2	0.25	4	–	–

*not detected.

Concerning the nanoemulsified essential oils, the antibacterial effectiveness was lower than essential oils, indicating that nanoemulsification does not necessarily improve the antimicrobial activity of essential oils (**Figure 5**). As previously mentioned, *C. citratus* essential oil was the most active; it was noted that the more highly effective the essential oil was, the more effective its nanoemulsion was; however, this does not necessarily apply to all the essential oils. It is worth shedding light on the resistance of *P. aeruginosa* to all the nanoemulsions. For *Salmonella*, *C. citratus* nanoemulsion was active only at 15 μL (*P* > 0.05), with inhibition zones of 9.67 ± 0.58 mm. For *E. coli*, the nanoemulsified *C. citratus* showed inhibition zones of 10 ± 1 mm and 10.67 ± 0.47 mm at 10 μL and 15 μL, respectively, and 9 ± 0 mm to 12.67 ± 0.58 mm against *S. aureus.* In contrast, it inhibits the growth of *B. subtilis* (*P* ≤ 0.05). On the other hand, *C. limon* nanoemulsion was inefficient against all the bacterial strains. Regarding the outcomes of the microdilution method, *C. citratus* nanoemulsion exhibited strong activity compared to *C. limon* nanoemulsion, which was not active against all the strains (**Table 3**).

The MBC results ranged from 2% to 0.25% (v/v) for the free essential oils and from 4% to 0.5% (v/v) for *C. citrus* nanoemulsion (**Table 3**). These results demonstrated that the nanoemulsification process did not significantly enhance the bactericidal efficacy of the essential oils.

### 3.5 Antifungal effect

**Figure 6:**
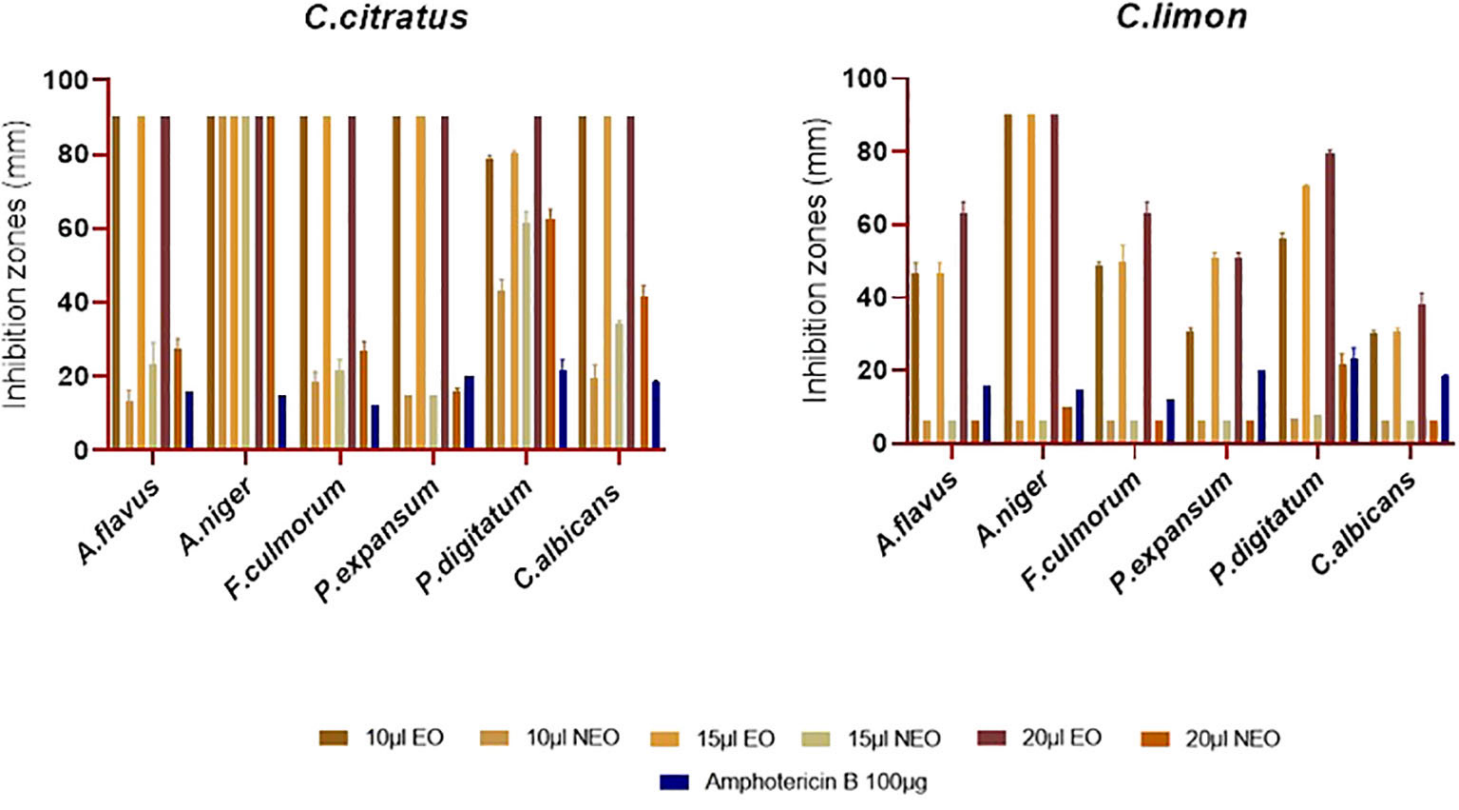
Antifungal activity of *C. citratus* and *C. limon* essential oils and their nanoemulsions using disk diffusion assay, with amphotericin B as the standard inhibitor, represented with error bars indicating ± standard deviation (*n* = 3).

The antifungal activity of the previous essential oils and their nanoemulsions was evaluated against the most important pathogenic fungi, including *A. niger*, *A. flavus*, *P. digitatum*, *P. expansum*, *F. culmorum*, and *C. albicans* (**Figure 6**). Notably, the essential oils and their nanoemulsions showed better antifungal activity than antibacterial activity. Consistent with the results obtained from the antibacterial assay, *C. citratus* essential oil had the highest level of potency, with complete inhibition of *A. niger*, *A. flavus*, *F. culmorum*, *P. expansum*, and *C. albicans*. Additionally, it showed very potent activity to total inhibition against *P. digitatum*, with inhibition zones ranging from 78.66 ± 1.15 mm to 90 ± 0.00 mm. Besides *C. citratus*, *C. limon* essential oil exhibited potential antifungal activity with inhibition zones spanning from 30.33 ± 0.57 mm to total inhibition (**Figure 6**).

The microdilution outcomes offered potent antifungal activity of the tested essential oils. *Cymbopogon citratus* displayed the highest activity, with MIC values of 0.03% (v/v) against both *P. digitatum* and *A*. *niger*. Meanwhile, it was 0.06% (v/v) against *F. culmorum*, *P. expansum*, and *C. albicans*. *Citrus limon* EO showed MIC values of 0.12% (v/v) against *P. expansum* and *C. albicans* and 0.25% (v/v) and 0.03% (v/v) against *A. flavus* and *P. digitatum*, respectively. The MIC value against *A. niger* and *F. culmorum* was 0.06% (v/v) (**Table 4**).

**Table 4:** MIC and MFC determination of *Cymbopogon citratus* and *Citrus limon* essential oils and their nanoemulsions against fungi.

Strain	*C. citratus* oil		*C. limon* oil		*C. citratus* nanoemulsion		*C. limon* nanoemulsion		
	MIC (%)	MFC (%)	MIC (%)	MFC (%)	MIC (%)	MFC (%)	MIC (%)		MFC (%)
*A. flavus* ATIM698	0.12	0.12	0.25	0.25	1	1	4		4
*A. niger* ATCC16888	0.03	0.06	0.06	0.06	0.25	0.25	4		4
*F. culmorum* KF91	0.06	0.06	0.06	0.06	0.12	0.12	4		4
*P. expansum*	0.06	0.06	0.12	0.12	0.12	0.12	0.12		0.12
*P. digitatum*	0.03	0.03	0.03	0.03	0.03	0.03	2		2
*C. albicans* ATCC 10231	0.06	0.06	0.12	0.12	0.12	0.12	–*		–

*not detected.

Concerning the results of the antifungal activity of the nanoemulsified essential oils, *C. citratus* displayed inhibition zones ranging from 15 ± 0 mm to 15.66 + 1.15 mm against *P. expansum*, the most resistant strain against the tested nanoemulsions. In addition, it showed inhibition zones ranging from 43.33 ± 2.88 mm to 62.66 ± 2.51 mm and from 19.66 ± 3.15 mm to 41.66 ± 2.88 mm against *P. digitatum* and *C. albicans*, respectively; additionally, it completely inhibited *A. niger*. The activity of the nanoemulsion of *C. limon* nanoemulsion was only active at 20 μL against *P. digitatum and A. niger* with inhibition zones of 21.66 ± 2.88 mm and 10 ± 0 mm, respectively. Notably, it was noted that the efficacy of *C. limon* nanoemulsion increased significantly with the increase in the dose (*P* ≤ 0.05). In contrast, no activity was exercised by *C. limon* nanoemulsion against *P. expansum*, *F. culmorum*, *A. flavus*, and *C. albicans* (**Figure 6**). Regarding the MIC values, *C. citratus* nanoemulsion was the most effective, showcasing MIC values of 0.12% (v/v) against *P. expansum*, *F. culmorum*, and *C. albicans*; values of 0.03% (v/v) and 0.25% (v/v) against *P. digitatum* and *A. niger*, respectively; and 1% (v/v) against *A. flavus*. *Citrus limon* nanoemulsion displayed a weaker activity against *A. flavus*, *A. niger*, and *F. culmorum*, showing values of 4% (v/v), with no activity against *C. albicans*. On the contrary, its activity was better against *P. expansum* and *P. digitatum*, with values of 0.12% (v/v) and 2% (v/v), respectively (**Table 4**).

On the other hand, the MFC outcomes were significantly varied, ranging from 0.25% to 0.03% (v/v) for the essential oils and from 4% to 0.03% (v/v) for their nanoemulsions (**Table 4**). This variation suggests that nanoemulsification might affect the fungicidal activity of the essential oils more than their bactericidal activity. Overall, the variability in MBC and MFC outcomes between free and nanoemulsified essential oils indicates that the nanoemulsification process may have differing impacts on their antimicrobial properties.

### 3.6 *In vivo* antifungal activity of the oil nanoemulsions

**Figure 7:**
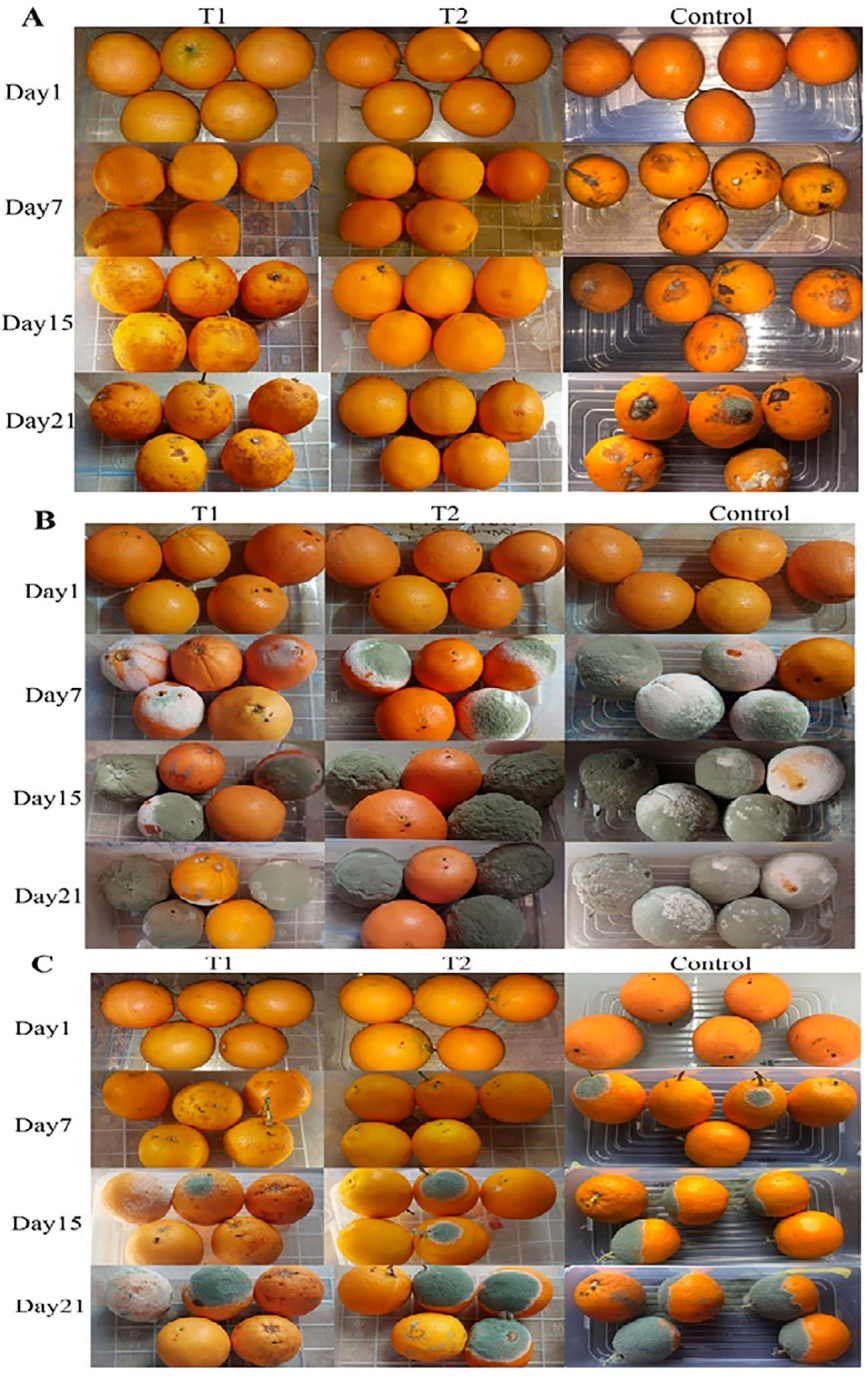
*In-vivo* antifungal activity of *C*. *citratus* (T1) and *C*. *limon* (T2) nanoemulsions during storage for 21 days **(A)**, after inoculating fruits with *P. digitatum* **(B)**, and after inoculating with *P. expansum* **(C)** compared to a control group.

Our study demonstrated the effectiveness of nanoemulsified *C. limon* and *C. citratus* coatings in preventing fungal growth on orange fruits. On the 7th day, no fungal growth was observed in the coated fruits, while the control group displayed slight mold occurrence and dark spots on the orange peel. On the 15th day, no signs of fungal growth were observed within the coated samples, except for slight drying, color alterations, and darkening in *C. citratus*-coated fruits (**Figure 7A**). On day 7, inoculated fruits with *P. digitatum* showed fungal growth in wounded spots of the coated fruits, with the control group showing a higher occurrence. On day 15, the coated samples exhibited fungus spread in the same oranges, while the control group was infected entirely. On day 21, no considerable difference was observed compared to day 15, indicating that the fungal progression remained constant (**Figure 7B**). Regarding the samples inoculated with *P. expansum*, on day 7, no growth was observed in the coated samples, with moderate growth in the control group. However, slight drying and color alterations were noticed on the fruit’s outer peel in samples coated with *C. citratus*. On day 15, the coated samples showed moderate *Penicillium* growth compared to the control. On day 21, the coated samples exhibited noticeable fungal proliferation within the fruit, with important dissemination in the control group (**Figure 7C**).

### 3.7 Assessment of fruit quality

**Figure 8:**
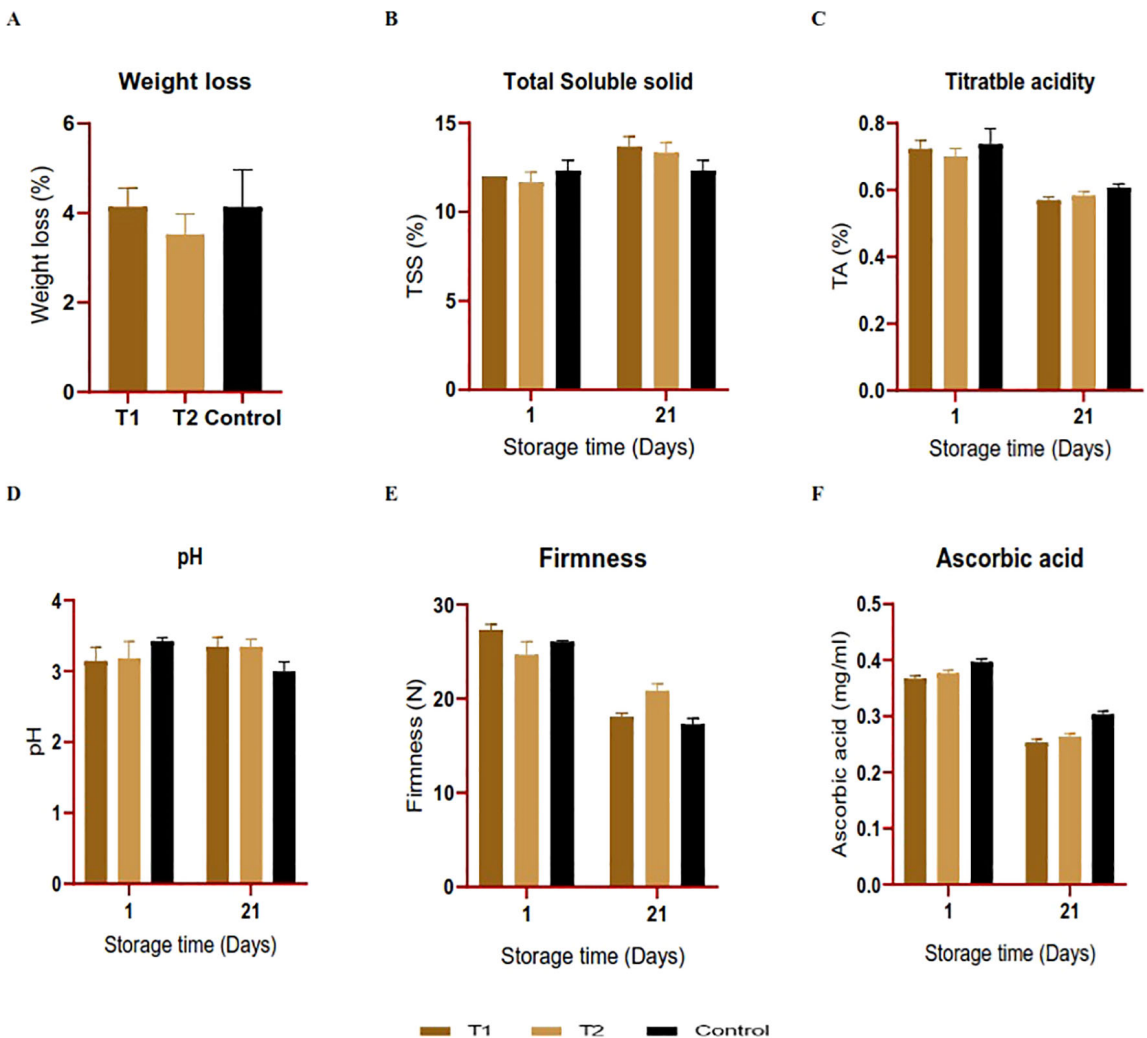
**(A–F)** The effect of nanoemulsions derived from *C. citratus* (T1) and *C. limon* (T2) on the quality parameters of orange fruits was studied over a 21-day storage period at room temperature. Error bars represent ± standard deviation (*n* = 3).

After a storage period of 21 days, differences in physicochemical parameters were observed between the coated and control orange fruits, as seen in **Figure 8**. The weight loss analysis outcomes conducted over 3 weeks at room temperature revealed a weight reduction within all the treatments accompanied by notable dehydration, with no significant differences (*P* > 0.05). The highest loss (4.13% and 4.14%) was recorded in the control and the fruits coated with *C. citratus* nanoemulsion; however, fruits treated with *C. limon* nanoemulsions displayed a relatively moderate weight loss, with values of 3.52%, respectively (**Figure 8A**). The TSS percentage varied among treatments and the control group. After 21 days of storage, the TSS level increased for the coated oranges, with 13.66°Brix and 13.33°Brix for the *C. citratus* and C. *limon* nanoemulsion-coated samples, respectively. However, no significant difference existed between treatments (*P* > 0.05), as shown in **Figure 8B**. The pH values exhibited no significant difference (*P* > 0.05) in all treatments, indicating a minor increase, with the most notable decrease in uncoated fruits (**Figure 8D**). The TA percentage declined in the fruits treated with *C. citratus* nanoemulsion, in comparison to those coated with *C. limon* nanoemulsion and the control group. Overall, there was no significant difference (*P* > 0.05) in TA % in all the treatments (**Figure 8C**). In all treatments, fruit firmness decreased with the advancement of the storage period (**Figure 8E**). On the day of the experimental setup, fruit firmness was between 24.65 and 27.30 N. On the 21st day after storage, fruit firmness decreased, with the highest firmness observed in *C. limon* nanoemulsion-coated samples (20.83 N), followed by *C. citratus* nanoemulsion (18.01 N) and the control fruits (17.26 N). In agreement with the above findings, the ascorbic acid content decreased significantly after the storage period for both treated and untreated fruits (**Figure 8F**).

### 3.8 Sensory evaluation

**Figure 9:**
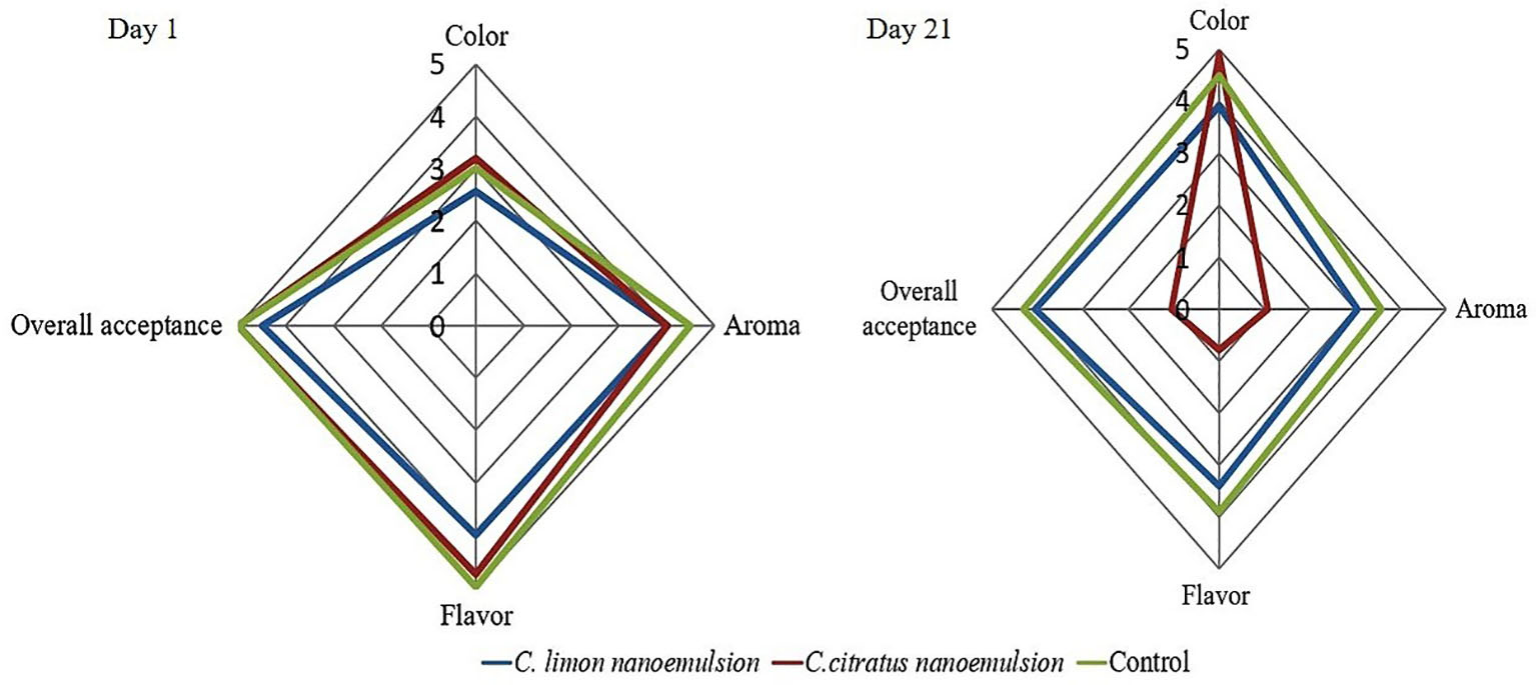
Radar chart illustrating the sensory characteristics of oranges on day 1 and after 21 days of storage. A rating system was employed, with color graded from pale yellow (0) to dark orange (5), aroma evaluated from weak (0) to strong (5), and overall acceptance rated from strong dislike (0) to strong liking (5).

The sensory evaluation of orange fruits was evaluated on the first day (day 1) and the last day of storage (day 21). The results revealed no significant differences between the coated samples and the control group on the first day of coating. In this study, the highest scores were achieved regarding color and aroma. On day 21, the control and coated samples with *C. limon* were the best, especially in texture, juiciness, and appearance, without significant differences, followed by fruits coated with *C. citratus* nanoemulsion. At the end of storage, all treatments slightly decreased the taste and flavor, especially in *C. citratus* nanoemulsion-coated samples, which displayed a darkening skin appearance, and an off-flavor, with alcoholic and fermented tastes. The nanoemulsions were acceptable except for *C. citratus* nanoemulsion (**Figure 9**; **Supplementary Figure S2**).

## 4 Discussion

Recent studies have found that aromatic plants like *C. citratus* and *C. limon* have antifungal effects in food, pharmaceutical, and other applications. Enhancing plant extracts using nanoformulations can improve their antifungal efficiency (Medeleanu et al., 2023). The yields of the current study agreed with previous research on *C. citratus* and *C. limon* essential oils (Himed et al., 2019; Medeleanu et al., 2023). The extraction method and environmental factors can affect the oil’s yield, quality, and composition. For example, *C. citratus* cultivated in Blida, Algeria, yielded 0.6% (Boukhatem et al., 2014), and the Lisbon variety of *C. limon* showed 0.81% (Himed et al., 2016).

The volatile profile of *C. citratus* oil in the current study is in line with Boudechicha et al. (2023), where neral, geranial, β-myrcene, geraniol, and geranyl acetate were the predominates but with some quantitative differences. For example, β-myrcene followed geranial and neral concentrations, which agreed with Blida oil (Benoudjit et al., 2022). On the other hand, limonene was the major component of *C. limon*, according to Himed et al. (2016) and Kehal et al. (2023). However, the dominance of neral, geranial, and γ-terpinene; the decline of β-pinene; and the presence of *p*-cymene represent the main differences for the first time in the literature (**Table 1**; **Figure 1**).

Despite the advantages of nanotechnology, the current study showed the effect of the intensive energy approach used as ultrasonication on the composition of essential oils investigated for the first time, which may change their volatile profile and, therefore, their bioactivity. In both oils, nanoemulsification leads to an increase of neral and geranial at the expense of monoterpenes, especially β-myrcene in *C. citratus* and limonene and γ-terpinene in *C. limon* (**Table 1**; **Figure 1**). The findings align with Boudechicha et al. (2023), who used microfluidization for homogenizing *C. citratus* oil with typical results. However, no information is available about the effect of homogenization on *C. limon* oil constituents. High-pressure homogenization of Algerian *Satureja hortensis* L. oil resulted in a significant increase in carvacrol content from 45.15% to 94.51%, along with a decline in γ-terpinene and *p*-cymene content (Boudechicha et al., 2024). It was found that γ-terpinene converts to carvacrol through aromatization and hydroxylation processes during storage of summer savory at different temperatures and times (Mohtashami et al., 2018).

The *C. citratus* and *C. limon* nanoemulsions have a mean particle size of 74.12 and 103 nm with a monomodal size distribution pattern, demonstrating an ultrafine size as it was less than 200 nm (**Supplementary Figure S1**). In a study by Dhanasekaran et al. (2024), the mean diameter of lemon oil–chitosan nanoemulsion was 130.01 nm, slightly higher than chitosan alone. The increase in diameter could be attributed to the concentration and encapsulation of lemon oil on the chitosan matrix. Similarly, the size of nanoemulsions could vary based on factors such as stabilizing agent, essential oil concentration, temperature, pH, agitation intensity, and ultrasonication time (Karimirad et al., 2019). Sabry et al. (2022) produced lemon oil emulsions ranging from 129 nm to 137 nm using Arabic gum and CMC as stabilizers. In another study, thyme oil nanoemulsion created by ultrasonically emulsifying with Tween80 showed a particle size of 83 nm (Patel and Ghosh, 2020). The PDI values ≤0.2 indicate uniformity among oil droplet sizes or monomodal distributions, as shown in **Supplementary Figure S2**, and therefore, better stability (Patel and Ghosh, 2020; Li et al., 2021). The ζ-potential is used to determine the electrical status of colloidal systems. In our study, the nanoemulsions showed a ζ-potential of ≥28 mV, indicating higher stability (Boudechicha et al., 2023). The non-ionic emulsifier Tween 80 gives oil droplets a negative charge, affecting the ζ-potential of emulsions containing different essential oils (Guerra-Rosas et al., 2016).

TEM was used to image nanoemulsification samples to examine their morphology (**Figure 2**). It has been reported in the literature that the particle sizes determined by the TEM were expected to be smaller than those specified by the DLS instrument because of the emulsion drying out in the air during preparation (Filippov et al., 2023). The same result was obtained by Aouf et al. (2020), who used the same technique to encapsulate Algerian *Saccocalyx satureioides* Coss. et Durieu, with well-dispersed round nanoparticles having a narrow size distribution.

Both orange fruit fungal isolates were *Penicillium* based on their color, consistency, conidiophores, and spores (**Figures 3**, **4**). For reliable fungal classification and identification, molecular biology methods must validate traditional findings (Yahya Allawi et al., 2022). The ITS sequence analysis showed that strain GY1 matched *P. expansum* (100% match), and GY2 matched *P. digitatum* (100% match). *Penicillium expansum* is not commonly found in citrus fruits. *Penicillium digitatum* and *P. italicum* are the main pathogens responsible for lesions on infected citrus fruits (Louw and Korsten, 2014). El-Dawy et al. (2021) showed the prevalence of *P. expansum* (38.2%) compared to other *Penicillium* species isolated from decaying citrus fruits. However, limited studies deal with the identification or resistance of such pathogens.

The antimicrobial activity of essential oils is influenced by their chemical composition and interactions between their constituents. Essential oils can impact membrane protein function and destroy bacterial structures, with higher doses having a greater effect on microorganisms (Khairan et al., 2024). The essential oil of *C. limon* exhibited weak to moderate activity against *P. aeruginosa*, *E. coli*, and *B. subtilis* and potential activity against *S. aureus*, in agreement with the literature (Aruna et al., 2022; Kehal et al., 2023). It also showed moderate to strong antibacterial activity against *K. pneumoniae*, in contrast to Louiza et al. (2018). This important activity is accepted due to the high percentage of oxygenated compounds and sesquiterpenes (Gupta, 2022). *Cymbopogon citratus* essential oil completely inhibited the growth of *S. aureus* and *B. subtilis* but exhibited weak to moderate activity against *P. aeruginosa*, in accordance with previous studies (Shendurse et al., 2021). Variations in the activity against Gram-negative bacteria may be related to their different thick outer membrane that can prevent the penetration of hydrophobic essential oils (Chao et al., 2000). It also showed lower activity against *K. pneumoniae* and *S. typhimurium* compared to a previous study (Mahmoud Mohamed et al., 2017). The activity against *E. coli* was good, similar to the study of Partovi et al. (2019). The strong antibacterial activity of *C. citratus* is expected due to its volatile components, including citral (Ujilestari et al., 2019).

With MIC values from 2% to 0.12%, the essential oils inhibited Gram-positive and Gram-negative bacteria (**Table 3**). This was more successful than disk diffusion, probably due to the essential oils’ solubility and diffusion in culture fluids and their volatility impacting paper disk dosages (Mangalagiri et al., 2021). In agreement with Partovi et al. (2019), *C. citratus* essential oil showed the lowest MIC values and the highest activity against all tested bacteria. The MIC values of *C. limon* essential oil were 2% to 0.5% against the tested bacteria, which are better than those obtained by Galgano et al. (2022).

The antibacterial effectiveness of the nanoemulsified essential oils was lower than that of free essential oils, except for *C. citratus* oil nanoemulsion against *B. subtilis*. The present study’s findings agreed with Jawaid et al. (2023), who reported good activity against *S. aureus* with a diameter of inhibition of 19.3 mm. Nanoemulsions can offer benefits like increased dispersion and stability. However, they may not always improve the antimicrobial activity of essential oils due to factors such as low oil concentration, physicochemical properties, and limited incubation time (Boudechicha et al., 2024). The results obtained in the current study indicated that *C. limon* nanoemulsion was not active, contrary to those of Yazgan et al. (2019), who found moderate to good activity against *K. pneumoniae*, *Salmonella paratyphi*, and *S. aureus*, respectively. This dissimilitude in results could be attributed to many factors, including different strains tested and differences in the methodology used.

Essential oils can inhibit fungal growth and control mycotoxin production simultaneously (Boudechicha et al., 2023, 2024). The complete inhibition of the examined fungi by *C. citratus* oil is similar to previous results (Helal et al., 2006; Boukhatem et al., 2014). The microdilution outcomes offered potent antifungal activity of the tested essential oils. *Cymbopogon citratus* displayed the highest activity, which is similar to previous findings, where an MIC value of 0.06% (v/v) against *A. niger* was found (Bansod and Rai, 2008). The MIC value against *F. culmorum*, *P. expansum*, and *C. albicans* was 0.06% (v/v). *Citrus limon* oil also showed an intense activity toward the investigated fungi, more potent than recorded against *A. niger*, *A. flavus*, and *P. chrysogenum* (Viuda-Martos et al., 2008).

The nanoemulsion of *C. citratus* showed complete inhibition of *A. niger*, a potential activity against *P. digitatum* and *C. albicans*, and moderate activity against *P. expansum*. On the other hand, *C. limon* nanoemulsion exhibited moderate inhibition of *P. digitatum*, very weak activity against *A. niger*, and no activity toward the other tested fungi (**Figure 6**). Regarding the MIC, the essential oils and their nanoemulsions showed activity against *P. digitatum* and *P. expansum*. This opens prospects to use the investigated oils’ nanoemulsions with all the encapsulation advantages as biopreservatives for citrus fruits. Some studies have shown contradictory results. In some cases, using a nanoemulsion seemed to lower the antimicrobial activity compared to the non-encapsulated compounds, while other studies showed higher antimicrobial activity of oils when using nanoemulsions (Cofelice et al., 2021).

The *P. expansum* and *P. digitatum* molds were greatly decreased by covering orange fruits with nanoemulsified *C. limon* and *C. citratus* (**Figure 7**). The control fruits showed dehydration, dryness during storage, and brown spots (Motamedi et al., 2018). Environmental variables pre- and post-harvest may darken and brown citrus fruit cells due to stress or injury. Finding the reasons for these defects might be challenging due to several considerations. Unlike coated samples, the control group had similar fungal development with minimal color changes (Zacarias et al., 2020). The control oranges infected with *P. digitatum* decayed the most after 7 days of storage compared to those treated with chitosan containing Denak, anise, and caraway essential oils (Aminifard and Bayat, 2018).

Current nanoemulsion coating research has shown considerable improvement in fruit quality (**Figure 8**). The barrier qualities of essential oils in coatings and their antioxidant activity decreased weight loss and gas and water interaction between fruit surfaces and the external environment (Shehata et al., 2020). After 21 days of storage, Khorram and Ramezanian (2021) found that cinnamon-oil-treated oranges lost more weight than controls. The research suggests that polysaccharide degradation into soluble sugars may impact TSS levels and fruit sweetness indicators during storage. Studies show that weight loss increases with sugar content, improving TSS, especially with particular essential oils (Aminifard and Bayat, 2018). The little rise in pH values, with the greatest reduction in uncoated fruits, corresponds with Radi et al. (2018), who found a slight increase in pH values during fresh-cut orange storage. The decreasing acidity and ascorbic acid concentration with time and increased fruit respiration may impact fruit acidity and pH (Wijewardane, 2022). Fruit respiration during lengthy storage has improved due to this organic acid decrease (Huang et al., 2021). *Cymbopogon citratus* nanoemulsion-coated fruits and the control group had a lower TA percentage than *C. limon*-coated samples, likely due to the interaction of the essential oil type and citrus fruit diversity, which significantly affect titratable acidity (Abdul-Rahaman et al., 2023). Nanoemulsions may postpone softening by improving pectin and enzyme activity and making the fruit resistant. Coating sweet oranges makes them firmer (Abdul-Rahaman et al., 2023).

Since essential oils are volatile compounds, their aroma will disappear with storage time. This study achieved the highest scores regarding color and aroma (**Figure 9**; **Supplementary Figure S2**). The choice of the appropriate dose of essential oil is a crucial factor since essential oils have a potent scent and flavor, which may negatively affect the organoleptic properties of the food product (Gurtler and Garner, 2022). On day 21, samples coated with *C. limon* nanoemulsion and the control were the same in texture, juiciness, and appearance, with no significant differences. These findings are similar to those of Gomes et al. (2017), who demonstrated that adding *C. limon* essential oil to the alginate coating did not affect the sensory properties of the fresh raspberries. At the end of storage, all treatments slightly decreased taste and flavor, especially in fruits coated with *C. citratus* nanoemulsion, which aligned with the findings of Khorram and Ramezanian (2021).

## 5 Conclusion

The Algerian essential oils of *Cymbopogon citratus* and *Citrus limon*, as well as their nanoemulsions prepared via ultrasonication, have shown promising results against both Gram-positive and Gram-negative bacteria as well as fungi identified in Thomson Navel oranges. When used as a coating on orange fruits inoculated with *P. expansum* and *P. digitatum* identified in fruits, the nanoemulsions significantly reduced fungal growth and minimized negative changes in fruit quality during storage. Intensive energy through ultrasonication successfully prepared particles on a nanoscale with good morphology and stability. However, it affects the oil constituents by increasing concentrations of oxygenated terpenes at the expense of non-oxygenated ones. The current study opens the perspective toward using available natural oils as preservatives for economic crops in developing countries like Egypt and Algeria.

## Data Availability

The datasets presented in this study can be found in online repositories. The names of the repository/repositories and accession number(s) can be found in the article/**Supplementary Material**.
