# Supplementary figures and images for: Antifungal effect of Algerian essential oil nanoemulsions to control Penicillium digitatum and Penicillium expansum in Thomson Navel oranges (Citrus sinensis L. Osbeck)
Source: Front Plant Sci. 2024 Nov 21;15:1491491. doi: 10.3389/fpls.2024.1491491 (PMC11617202; doi:10.3389/fpls.2024.1491491)

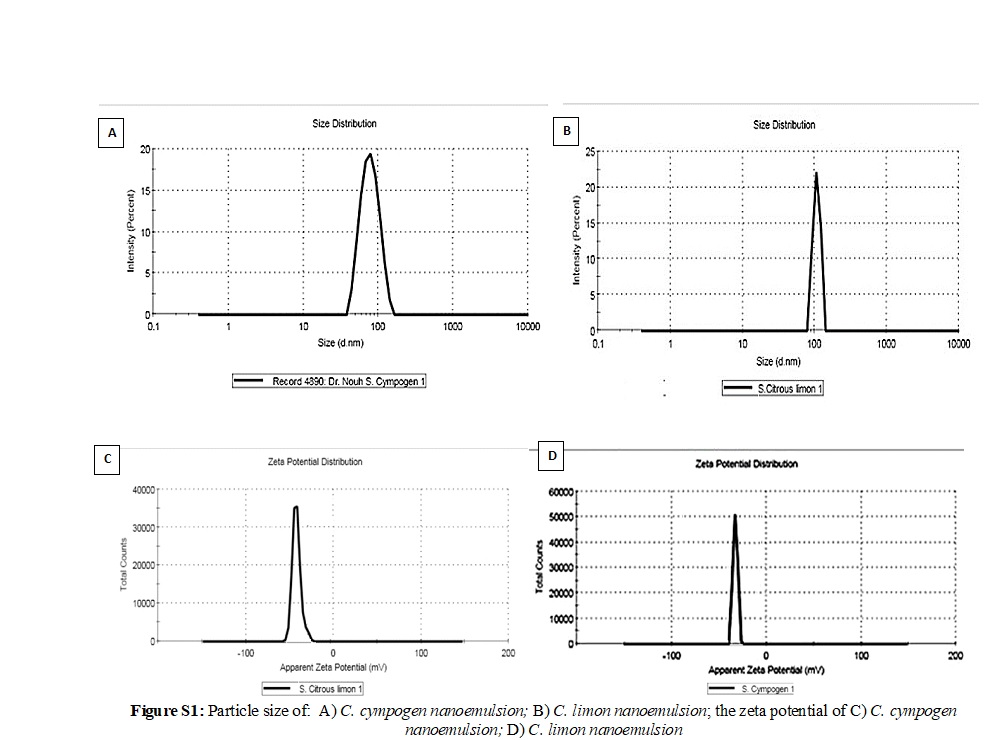

Supplement: Supplementary file 1 [file Image1.jpg]

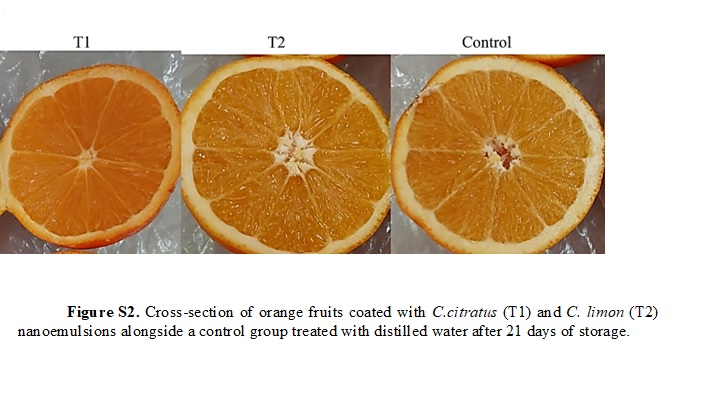

Supplement: Supplementary file 2 [file Image2.jpg]
